# Association of oxidative stress-related gst gene polymorphisms with periodontitis susceptibility: A systematic review and meta-analysis

**DOI:** 10.34172/japid.026.3866

**Published:** 2026-05-04

**Authors:** Paria Motahari, Negin Neshanifard

**Affiliations:** Deptartment of Oral and Maxillofacial Medicine, Faculty of Dentistry, Tabriz University of Medical Sciences, Tabriz, Iran

**Keywords:** GSTM1, GSTP1, GSTT1, Meta-analysis, Oxidative stress, Periodontitis, Polymorphism

## Abstract

**Introduction:**

Periodontitis is a prevalent inflammatory disease influenced by both genetic and environmental factors. Among genetic determinants, polymorphisms in oxidative stress-related g e n e s — p a r t i c u l a r l y *GSTM1*, *GSTT1*, and *GSTP1* (rs1695)—have been suggested as contributors to disease susceptibility. This systematic review and meta-analysis aimed to evaluate the association between *GSTM1*, *GSTT1*, and *GSTP1* (rs1695) gene polymorphisms and the risk of periodontitis.

**Methods:**

Following PRISMA 2020 guidelines, a comprehensive literature search was conducted in four databases (PubMed, Scopus, Web of Science, and Google Scholar) from inception to October 2025 for eligible case‒control studies. Pooled odds ratios (ORs) and 95% confidence intervals (CIs) were calculated. Heterogeneity was assessed using I^2^ and Q tests (with P-values), and publication bias was evaluated using Egger’s test and funnel plots. Evidence certainty was assessed with GRADE.

**Results:**

Seven studies with 1,525 participants were included. The GSTM1-null polymorphism was significantly associated with periodontitis (OR=2.94, 95% CI: 1.13–7.63, I2=93.5%) in subgroup analyses, particularly among Asians, in larger studies, and among heavy smokers. GSTT1-null polymorphism showed no significant effect (OR=0.64, 95% CI: 0.38–1.09, I^2^=69.7%). Initial pooled analyses indicated significant associations across all *GSTP1* (rs1695) genetic models; however, these associations were not robust and lost statistical significance after exclusion of a study that deviated from Hardy–Weinberg equilibrium. No publication bias was detected.

**Conclusion:**

This meta-analysis supports a potential role for the *GSTM1*-null polymorphism in increasing susceptibility to periodontitis. Associations for *GSTP1* (rs1695) were less consistent and sensitive to study quality, while the *GSTT1*-null polymorphism showed no clear effect. According to GRADE, the certainty of evidence was moderate for *GSTM1* and low for *GSTT1* and *GSTP1*; the results are therefore suggestive and require confirmation in larger multi-ethnic studies.

## Graphical Abstract

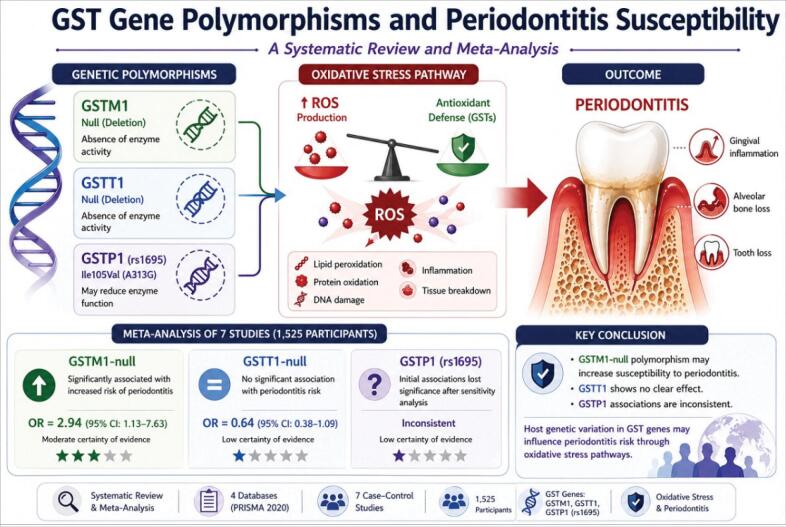

## Introduction

 Periodontitis is a complex, multifactorial inflammatory condition affecting the periodontal tissues, including the gingiva, periodontal ligament, and alveolar bone. If untreated, it leads to progressive attachment loss and eventual tooth loss, contributing significantly to the global burden of oral diseases.^1-3^ According to the 2017 World Workshop on the Classification of Periodontal and Peri-Implant Diseases and Conditions, the outdated terms “chronic” and “aggressive” periodontitis have been replaced by a unified classification system that stages and grades periodontitis based on severity, complexity, and risk of progression.^4^ This paradigm shift emphasizes the heterogeneous nature of periodontitis and highlights the necessity for a better understanding of its underlying biological mechanisms.^5-7^ While dental biofilm is the primary etiologic factor for periodontitis, not all individuals with similar levels of plaque accumulation exhibit the same disease severity. This discrepancy suggests a substantial role for host susceptibility, including genetic predispositions, in disease development and progression. Oxidative stress, caused by an imbalance between the production of reactive oxygen species (ROS) and the body’s antioxidant defenses, is a central contributor to periodontal tissue damage. Excessive ROScan induce lipid peroxidation, protein oxidation, and DNA damage, thereby promoting inflammation and tissue breakdown. Variations in the body’s antioxidant capacity may influence an individual’s susceptibility to oxidative stress-related periodontal injury.^8,9^ The glutathione S-transferase (*GST*) enzyme family is essential in detoxifying ROS and xenobiotic compounds. Functional polymorphisms in *GST* genes may impair detoxification capacity, increasing susceptibility to oxidative stress-related diseases.^8-10^ Three genetic polymorphisms have attracted particular attention in the context of periodontitis: *GSTM1*-null polymorphism, which results in a complete absence of the *GSTM1* enzyme; *GSTT1*-null polymorphism, which similarly leads to loss of *GSTT1* enzymatic activity; *GSTP1* rs1695 (A313G) single nucleotide polymorphism, which causes an isoleucine-to-valine (Ile105Val) substitution at codon 105, potentially affecting the enzyme’s substrate-binding capacity and function. Oxidative-stress pathways provide biological plausibility for the involvement of these polymorphisms in periodontal disease. The absence or reduced efficiency of *GST* enzymes may compromise detoxification of ROS-derived electrophilic compounds and disrupt redox homeostasis, thereby enhancing susceptibility to periodontal inflammation.^9-13^

 Multiple individual studies have examined the potential associations between *GSTM1*, *GSTT1*, and *GSTP1* (rs1695) polymorphisms and periodontitis,^14-20^ but their findings have been inconsistent due to small sample sizes, heterogeneous diagnostic criteria, and variations in population backgrounds. Although several narrative reviews have summarized the biological relevance of oxidative stress-related genetic factors in periodontal disease,^21,22^ to date, no systematic review and meta-analysis has jointly synthesized and quantitatively evaluated the available evidence on *GSTM1, GSTT1*, and *GSTP1* (rs1695) polymorphisms in periodontitis. Therefore, despite the existence of primary studies, a comprehensive meta-analysis has not yet been conducted, leaving important gaps regarding the overall magnitude and certainty of these associations. The present study addresses this gap by providing a comprehensive quantitative synthesis jointly examining *GSTM1, GSTT1*, and *GSTP1* (rs1695) polymorphisms in periodontitis. The review incorporates the GRADE framework to assess evidence certainty and applies a structured *PICOS* approach to enhance methodological transparency, reproducibility, and clinical relevance. Using a *PICOS* framework, we addressed: P — adults ( ≥ 18) with clinically/radiographically defined periodontitis; I — presence of *GSTM1*, *GSTT1,* or *GSTP1* (rs1695) variants; C — periodontally healthy controls; O — odds of periodontitis (OR, 95% *CI*); S — observational studies (case‒control/cross-sectional). A thorough evaluation of these polymorphisms could provide valuable insights into the genetic mechanisms underlying periodontitis, potentially informing prevention and treatment strategies.

## Methods

###  Study Design

 This systematic review and meta-analysis was conducted in accordance with the Preferred Reporting Items for Systematic Reviews and Meta-Analyses (PRISMA) guidelines. The review protocol was registered prospectively with PROSPERO (ID: CRD42025648483). Protocol preregistration was undertaken to enhance transparency and methodological rigor, in line with PRISMA 2020 recommendations.

###  Data Sources

 A comprehensive search strategy was designed to identify relevant articles published before October 2025. The following databases were systematically searched: Google Scholar, PubMed, Scopus, and Web of Science. The search strategy included a combination of Medical Subject Headings (MeSH) terms and free-text keywords:

 (" glutathione S-transferase” OR “*GSTM1*” OR “*GSTT1*” OR “*GSTP1*”) AND (“polymorphism” OR “genetic variant” OR “mutation”) AND (“periodontitis” OR “periodontal disease”)

 In line with common methodological practice in Google Scholar, only the first 200 records, sorted by relevance, were screened to balance feasibility and comprehensiveness. The exact database-specific search strategies for PubMed (MeSH + free-text terms), Scopus, and Web of Science—formatted according to each platform’s syntax—are provided in Supplementary File 1 to ensure full reproducibility and to address previous inconsistencies in search formatting. In addition to peer-reviewed full-text articles, grey literature sources (including one conference proceeding) were also included when they provided sufficient methodological detail and extractable genotype frequencies for quantitative synthesis. The inclusion of grey literature was prespecified to minimize potential publication bias, a recognized concern in genetic association studies with limited sample sizes.

 Reference lists of included studies and relevant reviews were manually screened to identify additional eligible publications. Two independent reviewers conducted the title/abstract screening and full-text assessment, with disagreements resolved through discussion. Duplicate records across databases were identified using automated tools, followed by manual verification to ensure accuracy.

###  Eligibility Criteria

 Studies were included if they: (1) were observational studies (case‒control, cohort, or cross-sectional) assessing *GSTM1*, *GSTT1*, or *GSTP1* (rs1695) polymorphisms; (2) enrolled adults aged ≥ 18 years; (3) reported genotype frequencies or sufficient data to calculate odds ratios (ORs) with 95% confidence intervals (CIs); (4) were full-text articles published in English; and (5) diagnosed periodontitis based on clinical and/or radiographic criteria. Studies were excluded if they: (1) lacked a periodontally healthy control group; (2) were reviews, editorials, case reports, or animal studies; or (3) did not provide sufficient genetic data or focused on other oral diseases.

###  Data Extraction

 Data were extracted independently by two reviewers (PM and NN) using a standardized data extraction form. The following information was collected: first author, year of publication, country, and ethnicity of the study population, study design, sample size, diagnostic criteria for periodontitis, genotyping method used, and smoking status. Disagreements between reviewers were resolved through discussion and consensus, and a third reviewer was available but ultimately not required.

###  Quality Assessment

 The quality assessment of included studies was performed using the Newcastle-Ottawa Scale (NOS) for cross-sectional and case-control studies. The NOS evaluates studies based on three broad perspectives: selection, comparability, and outcome assessment. Studies scoring ≥ 7 were considered high quality, 5–6 moderate quality, and ≤ 4 low quality. Risk-of-bias assessments, sensitivity analyses, and interpretation of pooled estimates were performed.

###  Hardy–Weinberg Equilibrium (HWE)

 HWE was tested in control groups for *GSTP1* (rs1695) using the chi-squared test. HWE was not applicable for *GSTM1* and *GSTT1* because these are gene-deletion polymorphisms (presence vs. null). HWE in controls was tested for *GSTP1* (χ^2^); studies with *P* < 0.05 were retained in primary analyses but examined in prespecified sensitivity analyses; any change in pooled estimates after exclusion was reported.

###  Data Analysis

 Statistical analyses were conducted using Comprehensive Meta-Analysis software (CMAversion 2). Pooled ORs and 95% CIs were calculated to assess the association between each GST polymorphism and periodontitis. Meta-analyses were performed using fixed-effect or random-effects models depending on heterogeneity, assessed by the I^2^ statistic and Cochran’s Q test. A random-effects model was applied when I^2^ > 50% or *P* < 0.10, as prespecified.

 Five genetic models for *GSTP1* (rs1695) were analyzed, including the allelic model (G vs. A), homozygous comparison (GG vs. AA), heterozygous model (AG vs. AA), dominant model (AG + GG vs. AA), and recessive model (GG vs. AG + AA). Prespecified subgroup analyses were conducted based on population size ( ≤ 100 vs. > 100), ethnicity (Asian vs. European), and smoking status (heavy smokers vs. light smokers) to explore potential sources of heterogeneity and biological differences, reduce the risk of post hoc data dredging, ensure methodological transparency, while avoiding data-driven selection of subgroups. Tunisia was analyzed separately where possible; due to the small African sample size and observed allele-frequency similarities with some Asian cohorts, we prespecified combining it with the Asian subgroup for exploratory analyses—this decision is acknowledged as a potential limitation. This approach aimed to improve statistical power and interpretability of ethnicity-specific effects.

 Sensitivity analyses were performed to evaluate the robustness of the results by omitting individual studies. Publication bias was assessed using funnel plots and Egger’s regression test (*P* < 0.05 indicating potential bias). GRADE certainty of evidence (risk of bias, inconsistency, indirectness, imprecision, publication bias) was independently evaluated by two reviewers, with disagreements resolved through consensus. The certainty of evidence was assessed using the GRADE framework, considering risk of bias, inconsistency, indirectness, imprecision, and publication bias. Language restriction to English publications was also considered a potential source of reporting bias and contributed to downgrading the certainty of the evidence for indirectness and publication bias.

## Results

###  Study Selection

**Figure 1:**
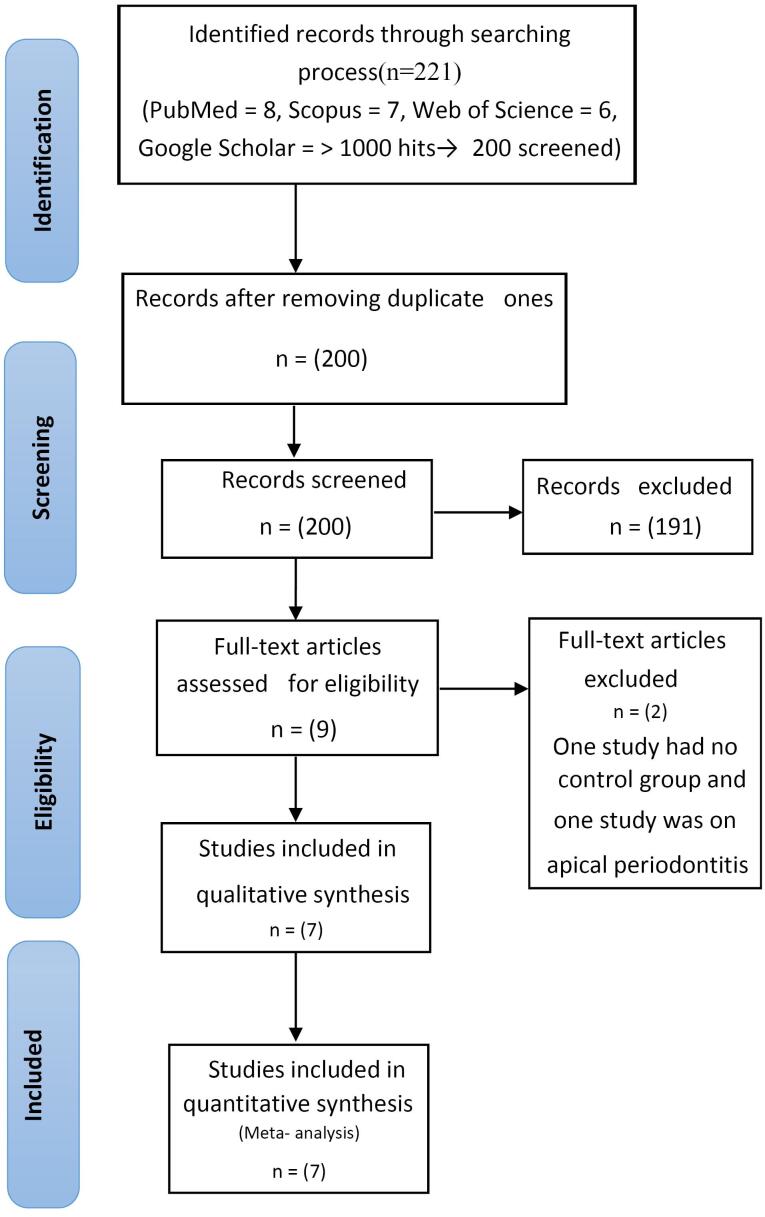
Study selection process (PRISMA 2020 flowchart)

 A total of 221 records were identified through database searching (PubMed = 8, Scopus = 7, Web of Science = 6, and the first 200 screened records from Google Scholar). After removing 21 duplicates, 200 unique articles remained for title and abstract screening. Based on the predefined eligibility criteria, 9 articles were selected for full-text assessment. Of these, two studies^23,24^ were excluded—one lacked a control group, and another did not meet the diagnostic criteria for periodontitis—resulting in 7 studies^14-20^ being included in the qualitative and quantitative synthesis (*GSTM1*: 6 studies; *GSTT1*: 5 studies; *GSTP1*: 3 studies), as presented in the PRISMA flow diagram (Figure 1).

###  Characteristics of Included Studies

Table 1 summarizes the characteristics of the 7 included studies. The total number of participants in these studies was 1525, with 752 periodontitis cases and 773 healthy controls. The included studies used polymerase chain reaction (PCR) methodology to assess the polymorphisms. All the studies had case‒control designs, with sample sizes ranging from 32 to 204 participants. The studies represented diverse populations from Asia, Europe, and North Africa, enhancing the generalizability of the findings. As Arshad et al.^19^ reported a significant deviation from HWE in controls (*P* = 0.001), we conducted sensitivity analyses with and without that study to evaluate its impact on the pooled estimates.

**Table 1:** Characteristics of included studies

**Authors**	**Year** **Country**	**Ethnicity**	**Case (M/F)**	**Control (n)**	**Age (year) in case and control**	**Smoking status**	**Genotyping method**	**HWE p**	**Location of polymorphism**
Saravanan et al.^14^	2023 Chennai-India	Asian	50(34/16)	50(34/16)	39 ± 8.2 41.3 ± 7.6	light-smokers	PCR	0.102	GSTP1
Concolino et al.^15^	2007 Italy	European	69(32/37)	61(27/34)	50.6 ± 8.2 34.4 ± 7.9	heavy smokers	PCR	NA	GSTM1,T1
Msolly et al.^16^	2015 Tunisia	African	80(45/35)	50(30/20)	39.78 ± 11.73 44.84 ± 12.68	light-smokers	PCR	NA	GSTM1,T1
Kim et al.^17^	2004 South Korea	Asian	115	126	-	heavy smokers	PCR	NA	GSTM1
Izakovicova et al.^18^	2024 Czech Republic	European	203(99/104)	204(108/96)	54.3 ± 7.4 48.6 ± 6	light-smokers	PCR	0.264	GSTM1,T1,P1
Arshad et al.^19^	2023 Pakistan	Asian	203(105/98)	201(105/96)	20-70 21-69	heavy smokers	PCR	0.001	GSTM1,T1,P1
Resende et al.^20^	2013 Portugal	European	32(16/16)	81(39/42)	44.9 ± 11.7 46.7 ± 10.67	light-smokers	PCR	NA	GSTM1,T1

PCR: Polymerase chain reaction; NA: not applicable; HWE: Hardy–Weinberg equilibrium p-value. Smoking categories were defined as heavy smokers ( ≥ 20 cigarettes/day) and light smokers ( < 10 cigarettes/day).

###  Meta-analysis Results

**Figure 2:**
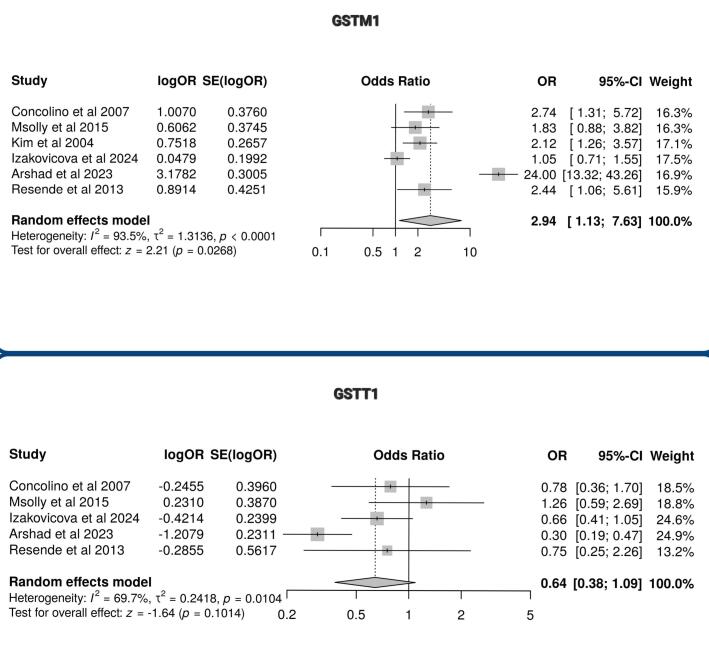
Forest plot of GSTM1 and GSTT1 null genotypes and periodontitis risk. The pooled odds ratio (OR) for GSTM1 was 2.94 (95% CI: 1.13–7.63, *P* = 0.03), indicating that individuals with the null genotype had nearly three-fold higher odds of periodontitis than carriers. Sensitivity analysis excluding an outlier study did not change the significance of the association. In five studies on GSTT1, the pooled OR was 0.64 (95% CI: 0.38–1.09; *P* = 0.10), suggesting no significant association between the GSTT1-null genotype and periodontitis susceptibility

 Six studies (712 cases/732 controls) assessed the association between the *GSTM1*-null polymorphism and periodontitis. The pooled analysis revealed a significant association, with individuals carrying the *GSTM1*-null genotype showing increased susceptibility to periodontitis (OR = 2.94, 95% CI: 1.13–7.63, *P* = 0.03) (Figure 2). Sensitivity analysis for *GSTM1*, excluding the study with an extreme odds ratio (Arshad et al.^19^), did not materially change the results, supporting the robustness of the findings (Supplementary File 2).

**Figure 3:**
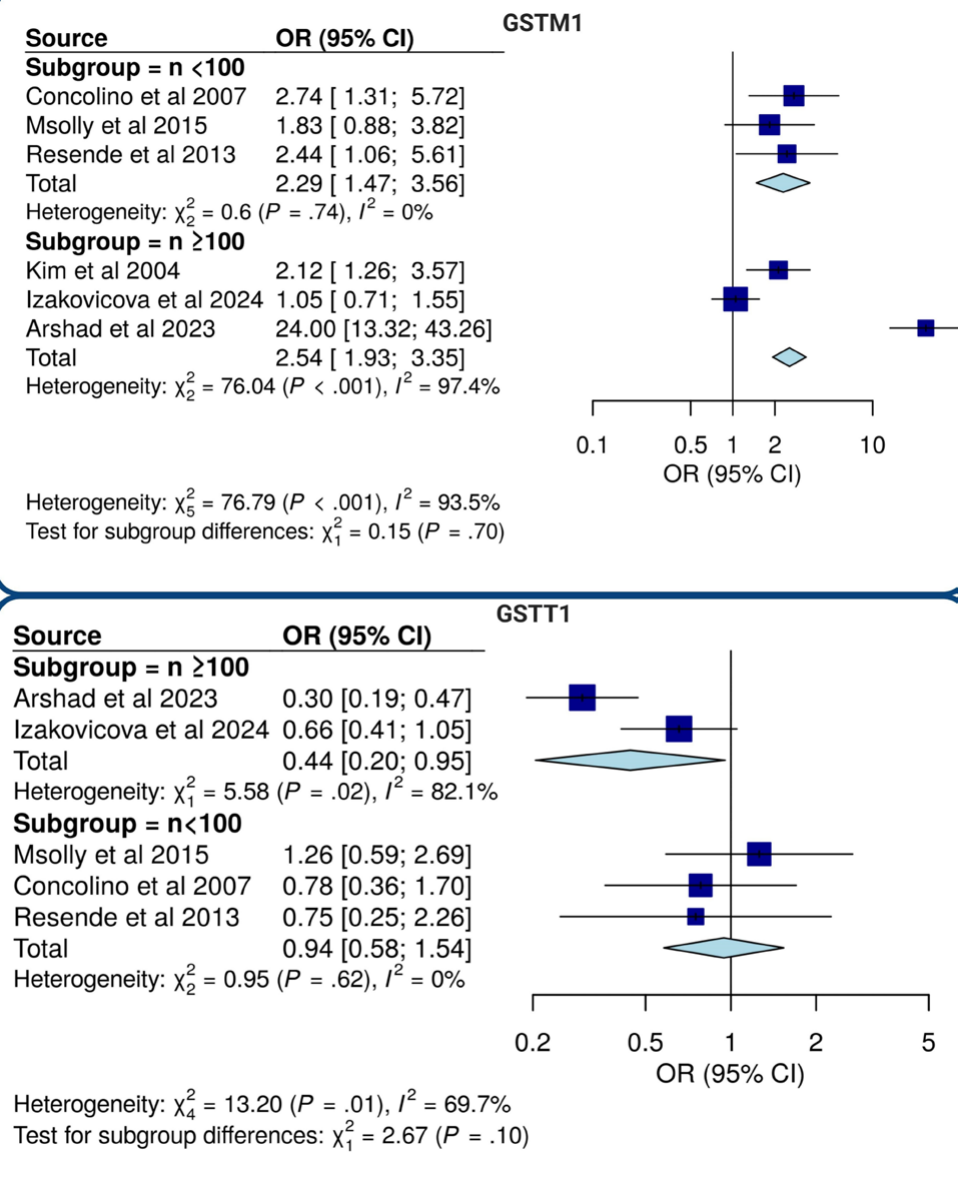
Subgroup analysis of GSTM1 and GSTT1 null genotypes by sample size. For GSTM1, stronger associations were observed in larger sample studies (n ≥ 100), whereas smaller studies (n < 100) showed weaker associations. GSTT1 results remained consistently non-significant across both strata

**Figure 4:**
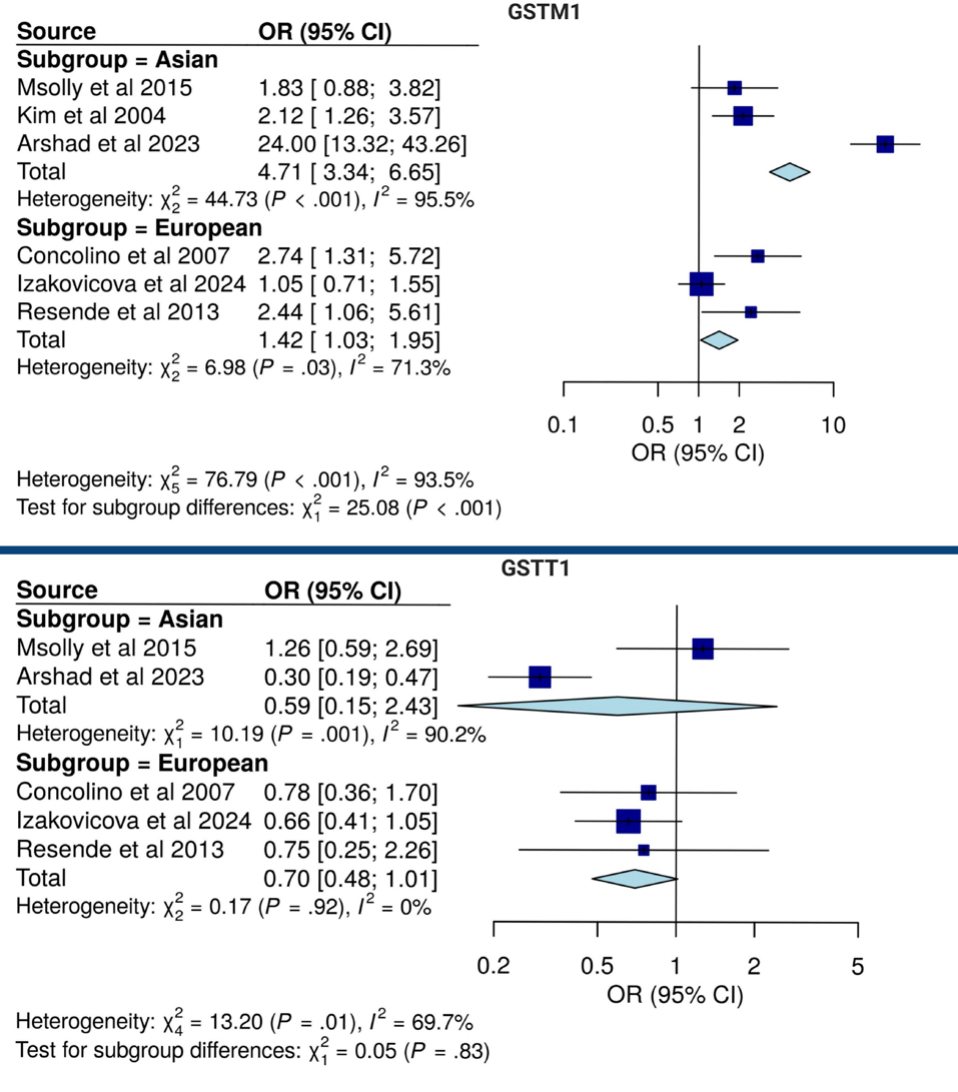
Ethnicity-based subgroup analysis of GSTM1 and GSTT1 null genotypes. Among Asian populations, the GSTM1-null genotype showed a stronger association with periodontitis (pooled OR ≈ 4.7) than in European populations (pooled OR ≈ 1.4; significant). The GSTT1-null genotype did not demonstrate significant associations in either ethnic subgroup

**Figure 5:**
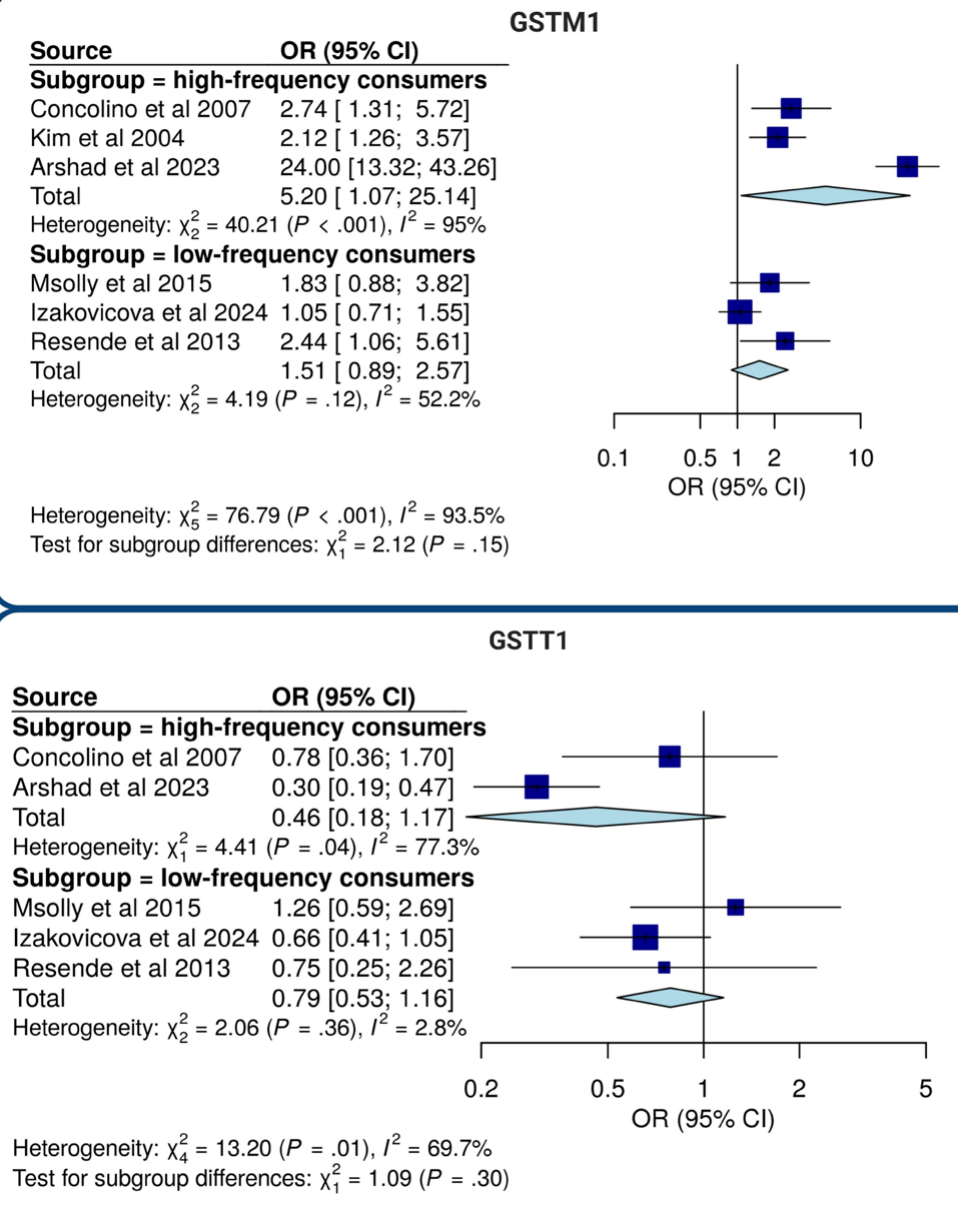
Subgroup analysis of GSTM1 and GSTT1 polymorphisms by smoking frequency: In heavy smokers, the GSTM1-null genotype was strongly associated with periodontitis (pooled OR > 5.0, significant), whereas light or non-smokers showed weaker or non-significant associations. The GSTT1-null genotype did not exhibit a consistent smoking-related effect

 Subgroup analyses were performed to explore the heterogeneity observed in the *GSTM1* analysis. The association between the *GSTM1*-null genotype and periodontitis was stronger in studies with sample sizes > 100 participants, indicating higher statistical power in larger cohorts (Figure 3). Studies conducted in Asian populations revealed a more pronounced association compared to European populations, likely reflecting genetic and environmental differences (Figure 4). Additionally, subgroup analysis based on smoking status showed a significantly stronger association in heavy smokers (Figure 5). This suggests that gene‒environment interactions, particularly tobacco-induced oxidative stress, may increase susceptibility to periodontitis in individuals with the *GSTM1*-null genotype.

 Five studies (645 cases/660 controls) examined *GSTT1*. For *GSTT1* polymorphisms, the meta-analysis did not exhibit a significant relationship with periodontitis (OR = 0.64, 95% CI: 0.38–1.09, *P* = 0.1) (Figure 2). Similarly, no statistically significant associations were observed in the subgroup analyses (Figures 3-5) or in the sensitivity analyses (Supplementary File 2). Taken together, these findings suggest that the *GSTT1* null genotype may not play a significant role in the pathogenesis of periodontitis.

**Figure 6:**
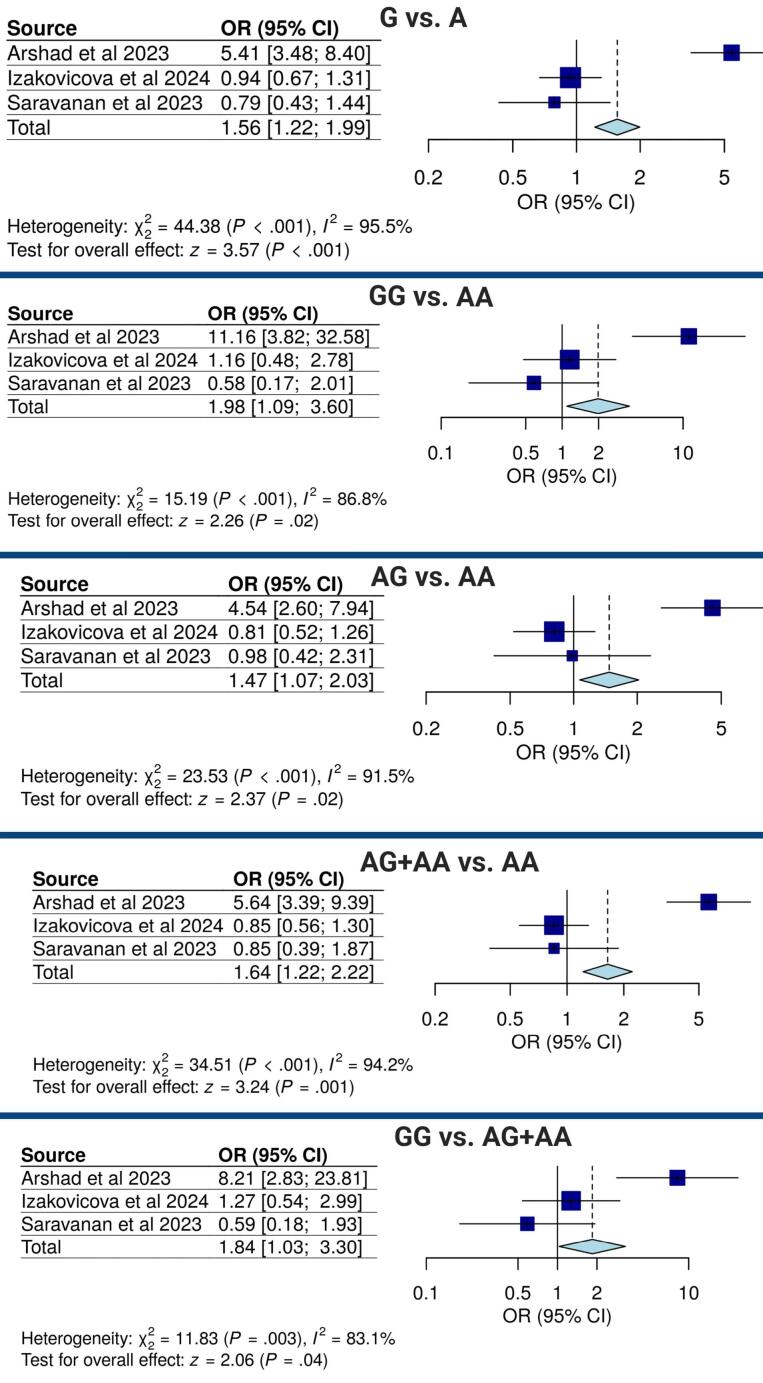
Genetic model-based analysis of GSTP1 polymorphism and periodontitis risk. Significant associations were found in all models: allelic (G vs. A, OR = 1.56), homozygous (GG vs. AA, OR = 1.98), heterozygous (GA vs. AA, OR = 1.47), dominant (GA + GG vs. AA, OR = 1.64), and recessive (GG vs. GA + AA, OR = 1.84). These findings suggest that the G allele confers increased susceptibility to periodontitis. However, these associations were not robust and lost statistical significance after exclusion of a study deviating from Hardy–Weinberg equilibrium

 Three studies (502 cases/498 controls) assessed *GSTP1* (rs1695) across five genetic models. A significant association was identified across all five genetic models of GSTP1 polymorphisms, with the G allele associated with an increased risk of periodontitis in each comparison (G vs. A, GG vs. AA, GA vs. AA, autosomal dominant, and autosomal recessive) (Figure 6). These findings support the hypothesis that the GSTP1 G allele may contribute to heightened susceptibility to periodontitis, potentially through mechanisms involving oxidative stress and regulation of inflammation. However, when the study by Arshad et al.^19^ (control HWE* P* = 0.001) was excluded in the sensitivity analysis (Supplementary File 2), the pooled associations lost statistical significance. Notably, removing this HWEp deviating study also markedly reduced heterogeneity, indicating that it had a disproportionate influence on the overall estimates and that the *GSTP1* association should therefore be interpreted with caution.

###  Quality Assessment of Included Studies

 The quality of the included studies was generally high, with an average NOS score of 7.8, indicating good quality. Most studies showed strong selection criteria and well-defined genotyping methods. However, variability in the adjustment for confounding factors was observed across studies, potentially affecting the robustness of the results. Detailed quality assessment scores are presented in Table 2.

**Table 2:** List of the quality score based on NOS

**Authors**	**Selection**				**Comparability**		**Outcome**			**Total score**	**Quality**^z^
	**Case definition adequate**	**Representativeness of the cases**	**Selection of controls**	**Definition of controls**	Main factor*	**Additional factor**^y^	**Ascertainment of exposure**	**Same method of ascertainment for cases and controls**	**Non-response rate**		
Saravanan et al.^14^	+	+	+	+	+	+	-	+	+	8	Good
Concolino et al.^15^	+	+	+	+	+	+	-	+	+	8	Good
Msolly et al.^16^	+	+	+	+	+	+	-	+	+	8	Good
Kim et al.^17^	+	+	+	+	-	-	+	+	+	7	Good
Izakovicova et al.^18^	+	+	+	+	+	+	+	-	+	8	Good
Arshad et al.^19^	+	+	+	+	+	+	+	-	+	8	Good
Resende et al.^20^	+	+	+	+	+	+	-	+	+	8	Good

*Age was matched between 2 groups. ^y^Sex was matched between 2 groups. ^z^Good quality (score: > 7) and fair quality (score: 5‒7).

###  Heterogeneity 

 Significant heterogeneity (I^2^ > 50%) was observed across studies, suggesting that differences in study populations, methodologies, and confounding variables may have influenced the findings.

###  Publication Bias

**Figure 7:**
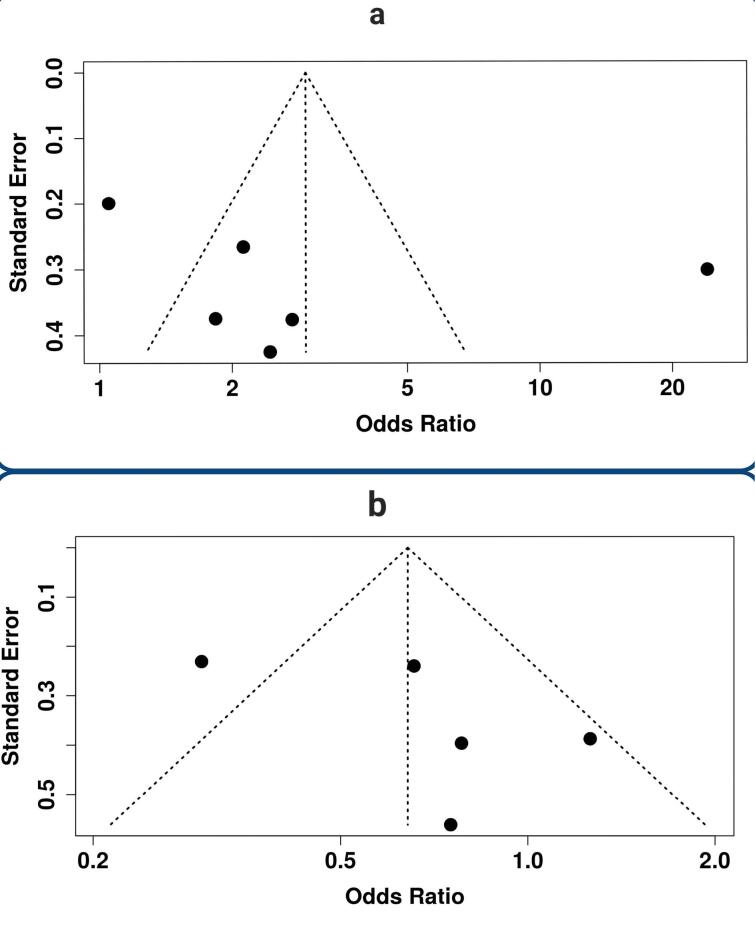
a: Funnel plot for publication bias in GSTM1-null polymorphism and periodontitis susceptibility (intercept: 5.11, 95% CI: -7.16 to 17.38, t: 0.817, *P*-value: 0.46). b: Funnel plot for publication bias in GSTT1-null polymorphism and periodontitis susceptibility (intercept: 3.47, 95% CI: -1.18 to 8.13, t: 1.463, *P*-value: 0.24)

 Visual inspection of funnel plots did not indicate substantial asymmetry (Figure 7). Egger’s regression test showed no evidence of publication bias for *GSTM1* (*P* = 0.46) and *GSTT1* (*P* = 0.24).

###  Certainty of Evidence

 According to the GRADE framework, the certainty of evidence was rated as moderate for the association between the *GSTM1*-null polymorphism and periodontitis. In contrast, the certainty of evidence was low for *GSTT1*-null and *GSTP1* (rs1695) polymorphisms. Downgrading decisions were primarily driven by imprecision related to small sample sizes and wide confidence intervals, the limited number of available studies, inconsistency across populations and diagnostic criteria, and potential reporting bias associated with restriction to English-language publications. The implications of these ratings—namely, limited confidence in the pooled effect estimates and the need for larger, methodologically robust studies—are summarized in the main Results section, with detailed GRADE judgments provided in Supplementary File 3.

## Discussion

 Periodontitis is strongly influenced by oxidative stress, and *GST* enzymes play essential roles in detoxifying ROS. In this meta-analysis, the *GSTM1*-null genotype showed a significant association with higher periodontitis susceptibility, whereas results for GSTT1 were inconclusive. Associations for *GSTP1* (rs1695) were sensitive to deviation from HWE, reducing confidence in this finding. Overall, these findings suggest that reduced antioxidant capacity may be associated with increased periodontal vulnerability, although causal inference is not possible due to the observational nature of the included studies.

 A key strength of this work is that, to our knowledge, it represents the first systematic review and meta-analysis to jointly examine *GSTM1, GSTT1*, and *GSTP1 *(rs1695) polymorphisms in periodontitis while incorporating GRADE-based certainty assessment. Previous literature consisted primarily of individual observational studies reporting inconsistent results, without quantitative synthesis. The present review extends the existing literature through PROSPERO registration, predefined eligibility criteria, structured risk-of-bias assessment, and evaluation of evidence certainty using GRADE.

*GST* enzymes detoxify electrophilic compounds, lipid peroxidation products, and regulate redox-sensitive signaling pathways. In periodontal tissues, dysbiotic biofilms and smoking increase *ROS* production, reduce glutathione levels, and activate *NF- κB* and *MAPK* pathways—conditions under which reduced *GST* activity may contribute to greater connective-tissue degradation.^25^ These mechanisms provide biological plausibility for the observed associations and for potential gene–environment interactions.

 The *GSTM1*-null genotype, which results in complete enzyme inactivity, was consistently associated with periodontitis across studies. Reduced *GSTM1* activity may impair detoxification of ROS, contributing to oxidative stress in periodontal tissues.^26-30^ Similarly, *GSTP1* (rs1695), which produces an Ile105Val substitution affecting enzyme efficiency and *MAPK* regulatory functions,^31-33^ showed initial associations across genetic models; however, these associations lacked robustness and lost statistical significance after exclusion of a study deviating from HWE. Accordingly, the available evidence for *GSTP1* (rs1695) should be interpreted as unreliable and non-robust, and any apparent associations are likely driven by methodological limitations rather than a stable genetic effect. By contrast, the *GSTT1*-null polymorphism was not significantly associated with periodontitis. This may reflect a more limited role of *GSTT1* in antioxidant defense, the presence of compensatory pathways, or insufficient statistical power in the included studies.^34,35^

 Subgroup analyses offered additional insight. The association between *GSTM1*-null and periodontitis was stronger in studies involving heavy smokers, Asian populations, and larger sample sizes. Smoking markedly increases oxidative stress, and heavy smokers experience greater ROSexposure than light or non-smokers.^36,37^ This may amplify the impact of reduced *GST* activity, supporting the presence of gene–environment interactions. Such findings indicate that smoking intensity should be considered when interpreting genetic risk in periodontitis.

 Meta-regression demonstrated a significant influence of sample size on effect magnitude, suggesting possible small-study effects. Smaller studies may overestimate associations due to reduced precision or publication bias. By incorporating sample size into the model and comparing results across strata, we were able to evaluate the stability of estimates and reduce the impact of small-study distortions.

 Consistent with the GRADE assessment, the association between the *GSTM1*-null genotype and periodontitis was supported by moderate-certainty evidence, whereas evidence for *GSTT1-*null and *GSTP1* (rs1695) polymorphisms remained of low certainty. These lower ratings were mainly attributable to imprecision, a limited number of studies, population heterogeneity, and the sensitivity of pooled estimates to methodological factors, such as deviations from HWE.

 Several limitations should be acknowledged. The number of eligible studies was small, particularly for *GSTP1*, and some had limited sample sizes. Variability in diagnostic criteria, ethnic composition, unmeasured confounders (e.g., diabetes, oral hygiene, and socioeconomic status), and restriction to English-language publications may have introduced bias. Including Tunisian data within the Asian subgroup, although justified by allele frequency similarities and the scarcity of African studies, may affect ethnicity-specific interpretations. *GSTP1 *findings were also sensitive to the exclusion of a single HWE-deviating study. Although Egger’s tests did not indicate publication bias, the small number of included studies limits the strength of these assessments. Restriction to English-language publications may have introduced reporting bias and limited the generalizability of the findings. This issue was explicitly considered in the GRADE assessment and contributed to downgrading the certainty of evidence for indirectness and potential publication bias.

## Implications for Practice

 Routine genetic testing for *GST *variants is not currently recommended. However, awareness of potentially elevated risk among *GSTM1*-null carriers—particularly heavy smokers—may inform hypothesis-generating considerations for future preventive strategies, such as smoking cessation counseling and closer periodontal monitoring, rather than immediate clinical implementation. Comparisons with other inflammatory and oxidative stress-related polymorphisms (e.g., IL-1, TNF-α, and MMP) suggest that *GST* variants contribute modestly to the overall genetic risk profile in periodontitis, but evidence remains insufficient for clinical translation. These implications should be interpreted cautiously, given the observational design of the included studies and the moderate certainty of evidence for* GSTM1*. These statements are exploratory in nature and are not intended to support clinical decision-making or risk-stratified prevention at this stage, but rather to highlight priorities for future research.

 Future research should prioritize large, multi-ethnic cohorts using standardized diagnostic criteria, evaluation of gene–environment interactions (particularly smoking), functional assays to clarify the biological impact of *GST *variants, and multi-gene or genome-wide approaches to determine whether *GST* polymorphisms interact with other oxidative stress-related genes in driving periodontal susceptibility.

## Conclusion

 This systematic review and meta-analysis indicates that the *GSTM1*-null polymorphism is associated with increased susceptibility to periodontitis with moderate certainty of evidence. In contrast, the current evidence regarding *GSTT1*-null and *GSTP1* (rs1695) polymorphisms remains of low certainty, precluding firm conclusions. Further well-designed, large-scale studies are required to strengthen the evidence base, particularly for *GSTT1* and *GSTP1.*

## Competing Interests

 The authors declare that they have no competing interests regarding authorship and/or publications of this paper.

## Data Availability

 The data that support the findings of this study are available from the corresponding author upon reasonable request.

## Ethical Approval

 Not applicable.

## Supplementary files

Supplementary File 1. Detailed search strategies for each database

Supplementary File 2. ensitivity analysis results

Supplementary File 3. GRADE evidence profiles
