# Supplementary material for: Association of oxidative stress-related gst gene polymorphisms with periodontitis susceptibility: A systematic review and meta-analysis
Source: J Adv Periodontol Implant Dent. 2026 May 4;18(3):153–62. doi: 10.34172/japid.026.3866 (PMC13437218; doi:10.34172/japid.026.3866)
Supplement: Supplementary file 1 [file japid-18-153-s001.pdf]

Motahari and Neshanifard et al, J Adv Periodontol Implant Dent 2026;18(3):153-S1
doi:10.34172/japid.026.3866
https://japid.tbzmed.ac.ir

Supplementary File 1. Search Strategy

Review Title: Association of GSTM1, GSTT1, and GSTP1 (rs1695) Gene Polymorphisms and Susceptibility to Periodontitis

Updated Search: October 30, 2025

Databases: PubMed (MEDLINE), Scopus, Web of Science Core Collection, Google Scholar

Languages: English

Population: Humans

Publication Type: Journal Article

Timeframe: From inception to October 30, 2025

Duplicate Removal: Automatic (EndNote/Mendeley) + manual verification

1. PubMed (MEDLINE via pubmed.ncbi.nlm.nih.gov)

Search run: October 30, 2025

```
(("Periodontitis"[Mesh] OR periodontitis[tiab] OR "periodontal
disease"[tiab])
AND
("Glutathione Transferase"[Mesh]
OR "Glutathione S-Transferase"[tiab]
OR GSTM1[tiab] OR GSTT1[tiab] OR GSTP1[tiab]
OR rs1695[tiab] OR "Ile105Val"[tiab])
AND
(polymorphism*[tiab] OR genotype*[tiab] OR variant*[tiab]))
```

Filters applied:

- Humans
- English
- Journal Article
- Publication dates: inception – 2025/10/30

Total retrieved: 8 records

---

2. Scopus (TITLE-ABS-KEY)

```
TITLE-ABS-KEY (periodontitis OR "periodontal disease")
```

```
AND
TITLE-ABS-KEY (GSTM1 OR GSTT1 OR GSTP1
OR "glutathione s-transferase"
OR rs1695 OR "Ile105Val")
AND
TITLE-ABS-KEY (polymorphism OR genotype OR variant)
```

---

3. Web of Science (WoS Core Collection)

```
TS=(periodontitis OR "periodontal disease")
AND
TS=(GSTM1 OR GSTT1 OR GSTP1
OR "glutathione s-transferase"
OR rs1695 OR Ile105Val)
AND
TS=(polymorphism* OR genotype* OR variant*)
```

Refined by:

- Document Type: Article
- Language: English

---

4. Google Scholar

Search expression used:

```
(periodontitis OR "periodontal disease")
(GSTM1 OR GSTT1 OR GSTP1 OR "glutathione s-transferase" OR rs1695 OR
Ile105Val)
```

Settings and screening:

- Years: 1990–2025
- "Include citations" = ON
- **First 200 results screened manually**
- Duplicates & irrelevant records removed

Rationale:
Google Scholar does not provide reproducible search counts; therefore, screening was limited to the first 200 results, consistent with systematic review guidelines.

---

5. Deduplication Procedure

- Automatic removal (EndNote X9 + Mendeley)
- Manual verification using:
  - Title
  - Authors

    - Year
    - Journal
    - DOI/PMID
- Final duplicate check during full-text screening
