# Supplementary material for: Association of oxidative stress-related gst gene polymorphisms with periodontitis susceptibility: A systematic review and meta-analysis
Source: J Adv Periodontol Implant Dent. 2026 May 4;18(3):153–62. doi: 10.34172/japid.026.3866 (PMC13437218; doi:10.34172/japid.026.3866)

Motahari and Neshanifard et al, J Adv Periodontol Implant Dent 2026;18(3):153-S2
doi:10.34172/japid.026.3866
https://japid.tbzmed.ac.ir

Supplementary file 2

Results of the sensitivity analysis for GSTM1 polymorphism after excluding the study listed below the figure:

Study name	Odds ratio	Lower limit	Upper limit	Z-Value	p-Value
Concolino et al	2.742	1.314	5.720	2.689	0.007
Msolly et al	1.833	0.879	3.824	1.616	0.106
Kim et al	2.117	1.256	3.568	2.815	0.005
Izakovicova et al	1.051	0.712	1.550	0.250	0.803
Resende et al	2.434	1.056	5.610	2.087	0.037
	1.807	1.206	2.708	2.867	0.004

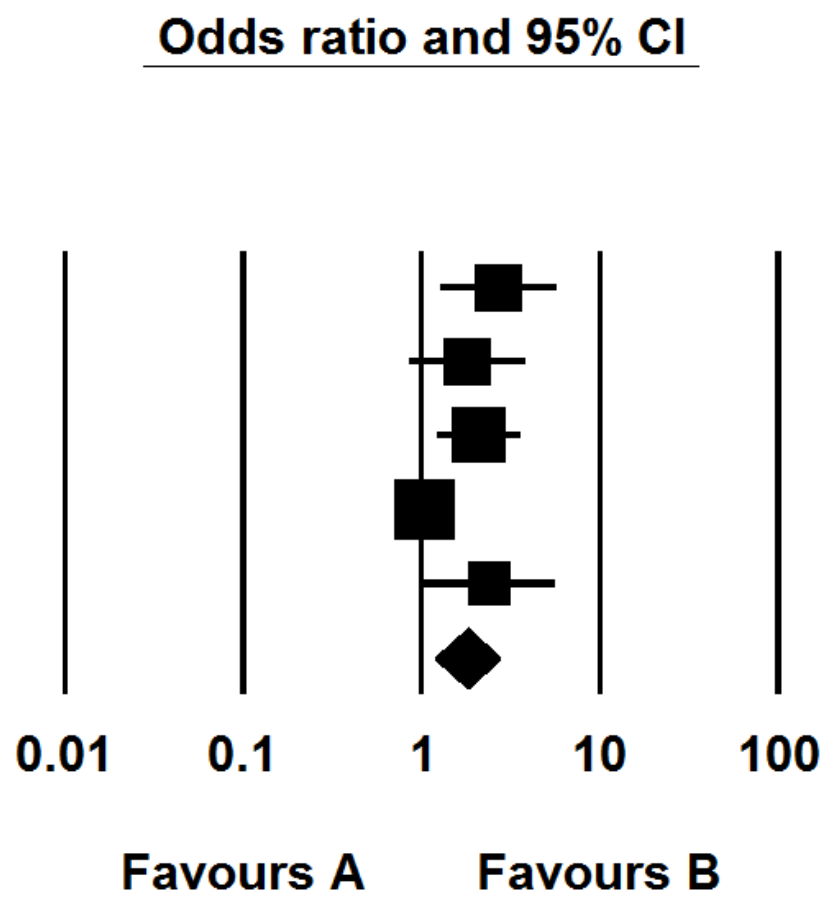

Arshad et al.[19]

Study name	Odds ratio	Lower limit	Upper limit	Z-Value	p-Value
Msolly et al	1.833	0.879	3.824	1.616	0.106
Kim et al	2.117	1.256	3.568	2.815	0.005
Izakovicova et al	1.051	0.712	1.550	0.250	0.803
Arshad et al	24.001	13.316	43.261	10.573	0.000
Resende et al	2.434	1.056	5.610	2.087	0.037
	2.434	1.902	3.115	7.071	0.000

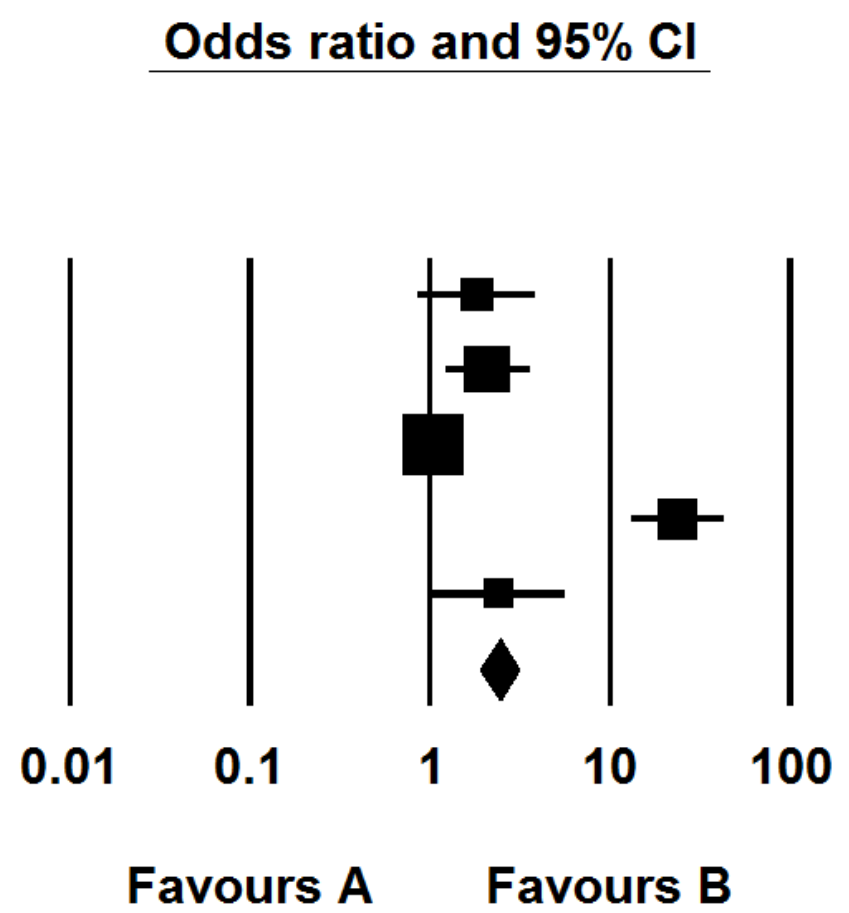

Concolino et al.[15]

Study name	Odds ratio	Lower limit	Upper limit	Z-Value	p-Value
Concolino et al	2.742	1.314	5.720	2.689	0.007
Msolly et al	1.833	0.879	3.824	1.616	0.106
Kim et al	2.117	1.256	3.568	2.815	0.005
Arshad et al	24.001	13.316	43.261	10.573	0.000
Resende et al	2.434	1.056	5.610	2.087	0.037
	3.658	1.303	10.268	2.463	0.014

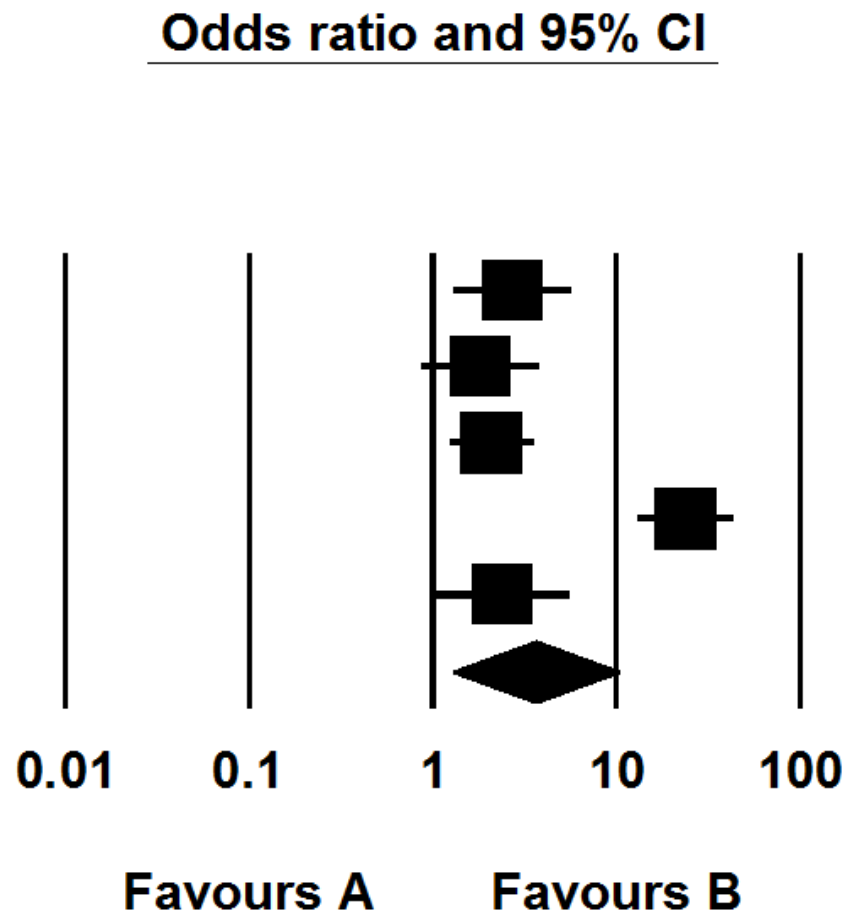

Izacovicova et al.[18]

Study name	Odds ratio	Lower limit	Upper limit	Z-Value	p-Value
Concolino et al	2.742	1.314	5.720	2.689	0.007
Msolly et al	1.833	0.879	3.824	1.616	0.106
Izakovicova et al	1.051	0.712	1.550	0.250	0.803
Arshad et al	24.001	13.316	43.261	10.573	0.000
Resende et al	2.434	1.056	5.610	2.087	0.037
	2.559	1.970	3.324	7.044	0.000

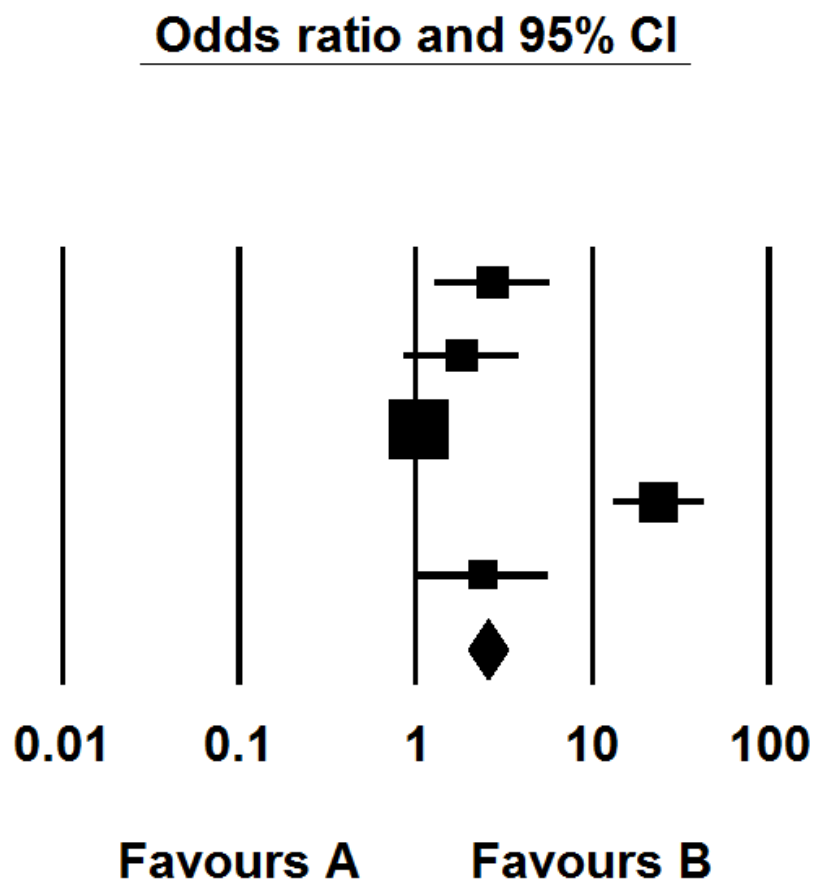

Kim et al.[17]

Study name	Odds ratio	Lower limit	Upper limit	Z-Value	p-Value
Concolino et al	2.742	1.314	5.720	2.689	0.007
Kim et al	2.117	1.256	3.568	2.815	0.005
Izakovicova et al	1.051	0.712	1.550	0.250	0.803
Arshad et al	24.001	13.316	43.261	10.573	0.000
Resende et al	2.434	1.056	5.610	2.087	0.037
	3.225	1.046	9.945	2.038	0.042

Statistics for each study

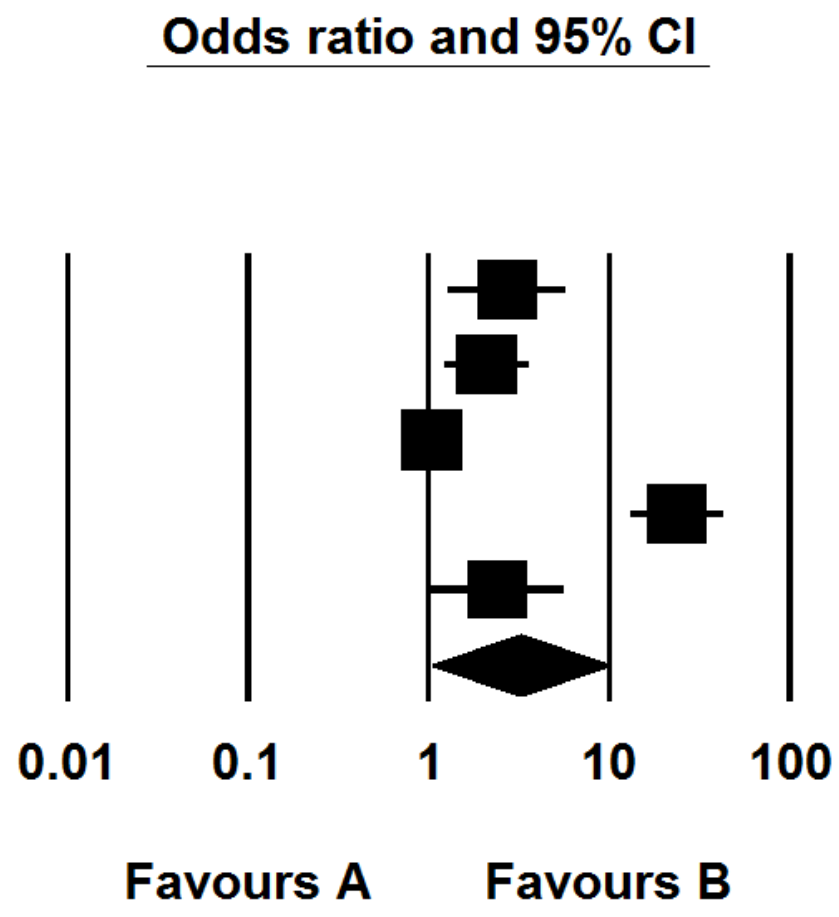

Msolly et al.[16]

Study name	Odds ratio	Lower limit	Upper limit	Z-Value	p-Value
Concolino et al	2.742	1.314	5.720	2.689	0.007
Msolly et al	1.833	0.879	3.824	1.616	0.106
Kim et al	2.117	1.256	3.568	2.815	0.005
Izakovicova et al	1.051	0.712	1.550	0.250	0.803
Arshad et al	24.001	13.316	43.261	10.573	0.000
	3.047	1.004	9.245	1.968	0.049

Statistics for each study

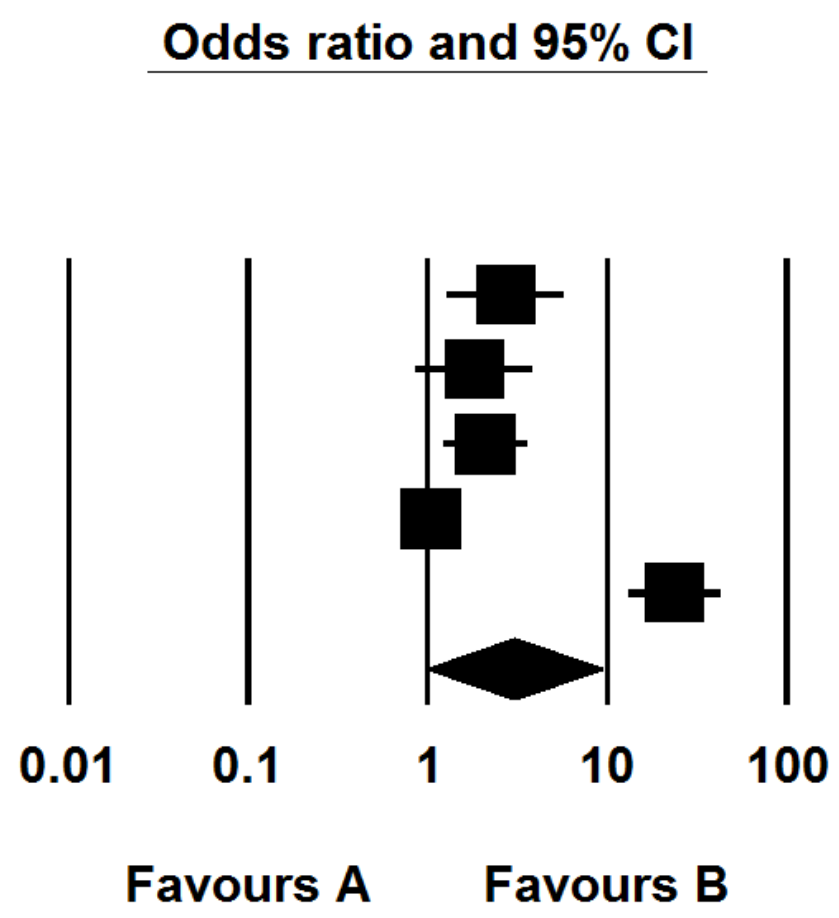

Resende et al.[20]

Results of the sensitivity analysis for GSTT1 polymorphism after excluding the study listed below the figure:

Study name	Odds ratio	Lower limit	Upper limit	Z-Value	p-Value
	Statistics for each study				
Concolino et al	0.781	0.359	1.697	0.624-	0.533
Msolly et al	1.256	0.588	2.685	0.589	0.556
Izakovicova et al	0.652	0.405	1.048	1.766-	0.077
Resende et al	0.752	0.250	2.260	0.507-	0.612
	0.780	0.555	1.096	1.430-	0.153

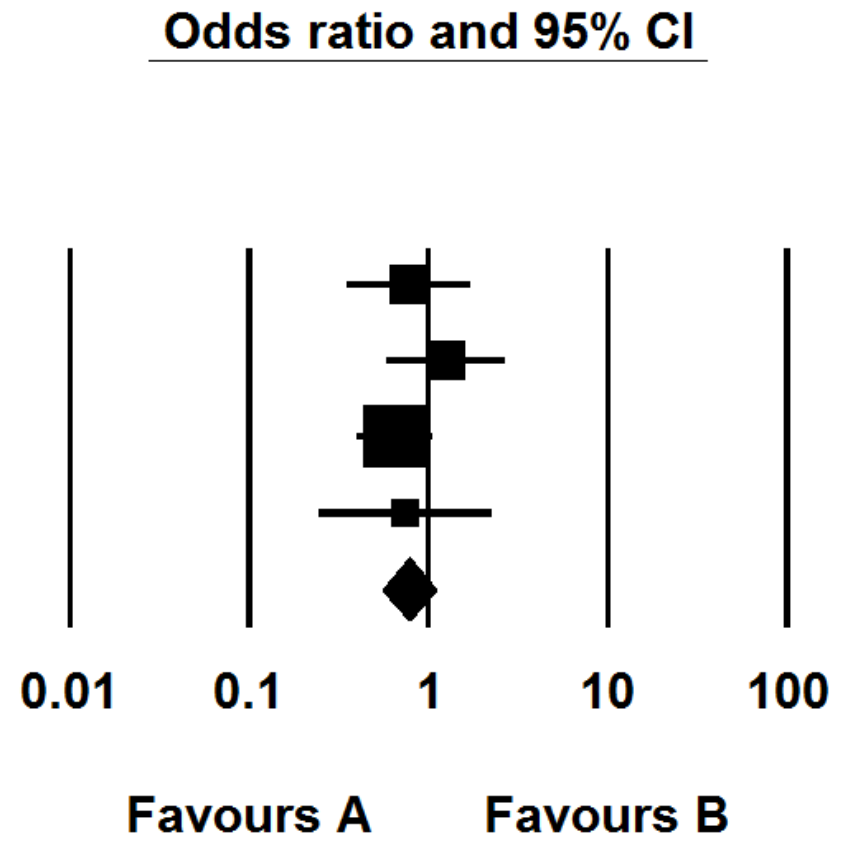

Arshad et al.[19]

Study name	Odds ratio	Lower limit	Upper limit	Z-Value	p-Value
	Statistics for each study				
Msolly et al	1.256	0.588	2.685	0.589	0.556
Izakovicova et al	0.652	0.405	1.048	1.766-	0.077
Arshad et al	0.294	0.185	0.468	5.178-	0.000
Resende et al	0.752	0.250	2.260	0.507-	0.612
	0.616	0.323	1.175	1.470-	0.142

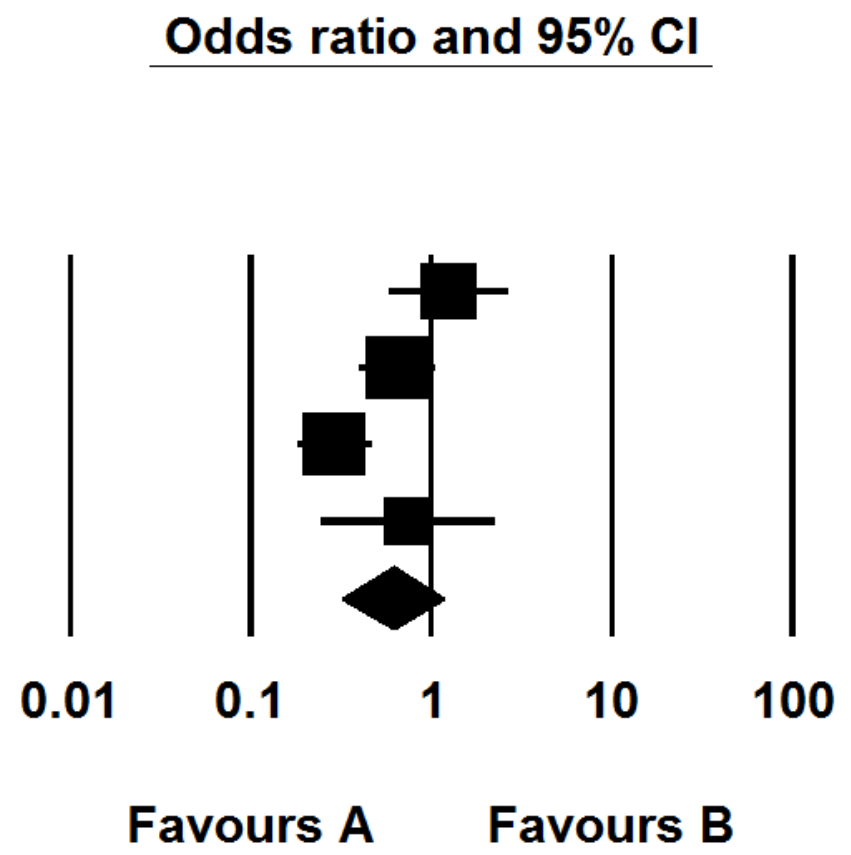

Concolino et al.[15]

Study name	Odds ratio	Lower limit	Upper limit	Z-Value	p-Value
	Statistics for each study				
Concolino et al	0.781	0.359	1.697	0.624-	0.533
Msolly et al	1.256	0.588	2.685	0.589	0.556
Arshad et al	0.294	0.185	0.468	5.178-	0.000
Resende et al	0.752	0.250	2.260	0.507-	0.612
	0.651	0.307	1.381	1.118-	0.263

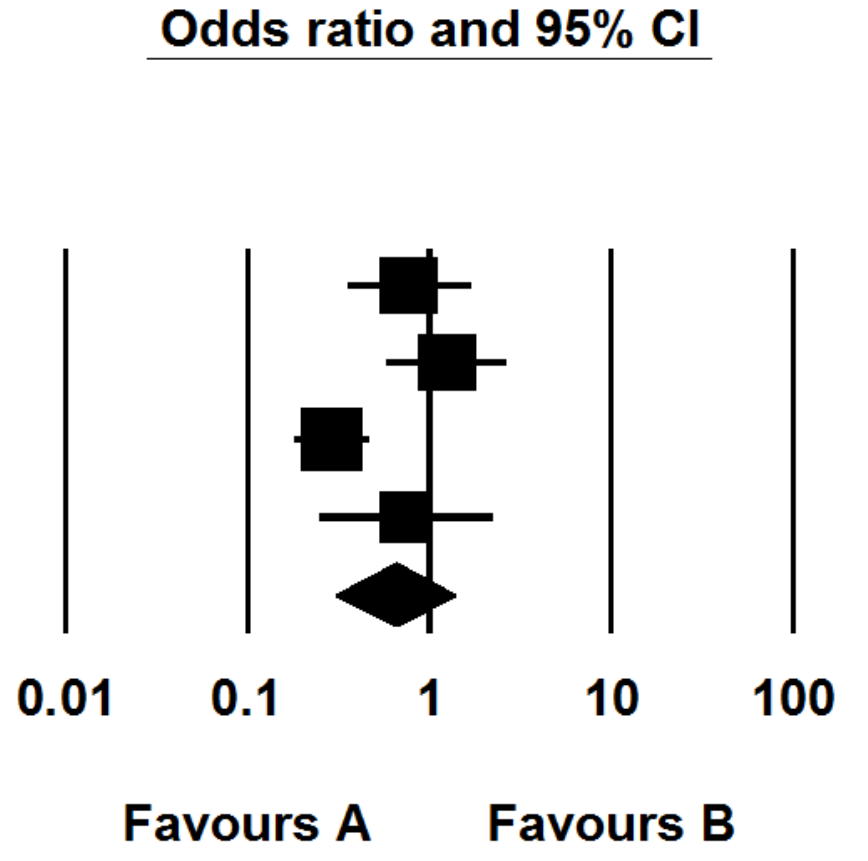

Izacivicova et al.[18]

Study name	Odds ratio	Lower limit	Upper limit	Z-Value	p-Value
	Statistics for each study				
Concolino et al	0.781	0.359	1.697	0.624-	0.533
Izakovicova et al	0.652	0.405	1.048	1.766-	0.077
Arshad et al	0.294	0.185	0.468	5.178-	0.000
Resende et al	0.752	0.250	2.260	0.507-	0.612
	0.538	0.318	0.908	2.319-	0.020

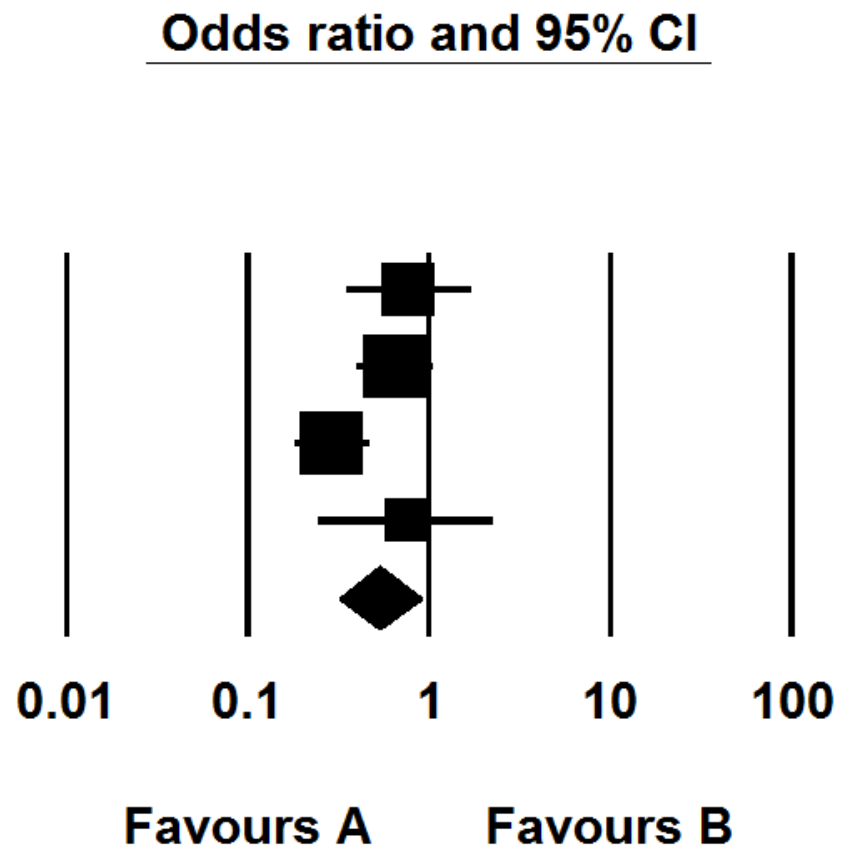

Msolly et al.[16]

Study name	Odds ratio	Lower limit	Upper limit	Z-Value	p-Value
Concolino et al	0.781	0.359	1.697	0.624-	0.533
Msolly et al	1.256	0.588	2.685	0.589	0.556
Izakovicova et al	0.652	0.405	1.048	1.766-	0.077
Arshad et al	0.294	0.185	0.468	5.178-	0.000
	0.627	0.339	1.159	1.490-	0.136

Odds ratio and 95% CI

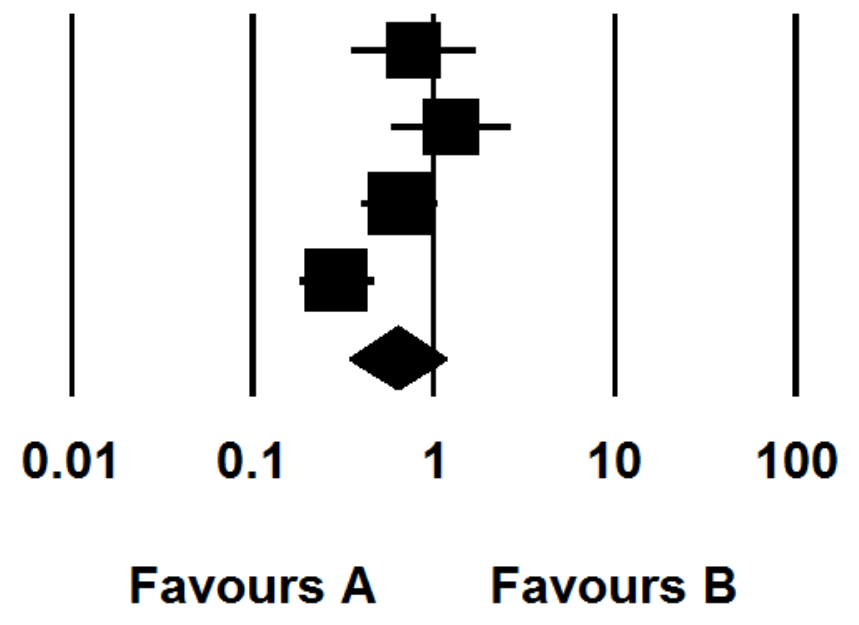

Resende et al.[20]

Sensitivity analysis of GSTP1 (rs1695) polymorphism under five genetic models after exclusion of the study by Arshad et al.[19]:

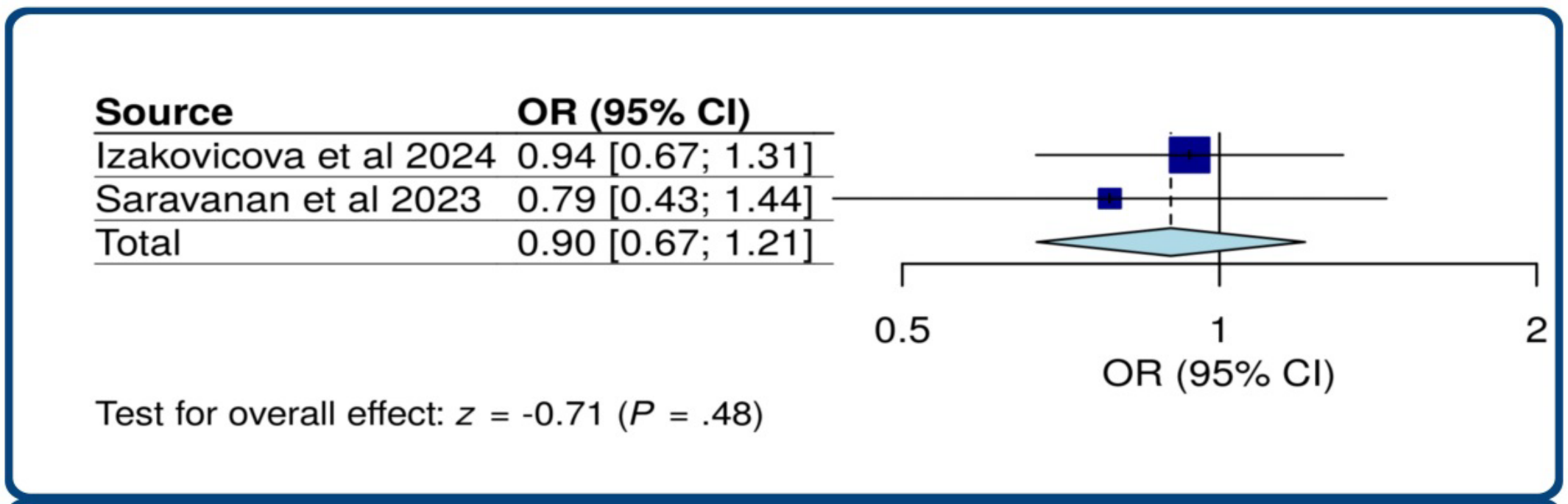

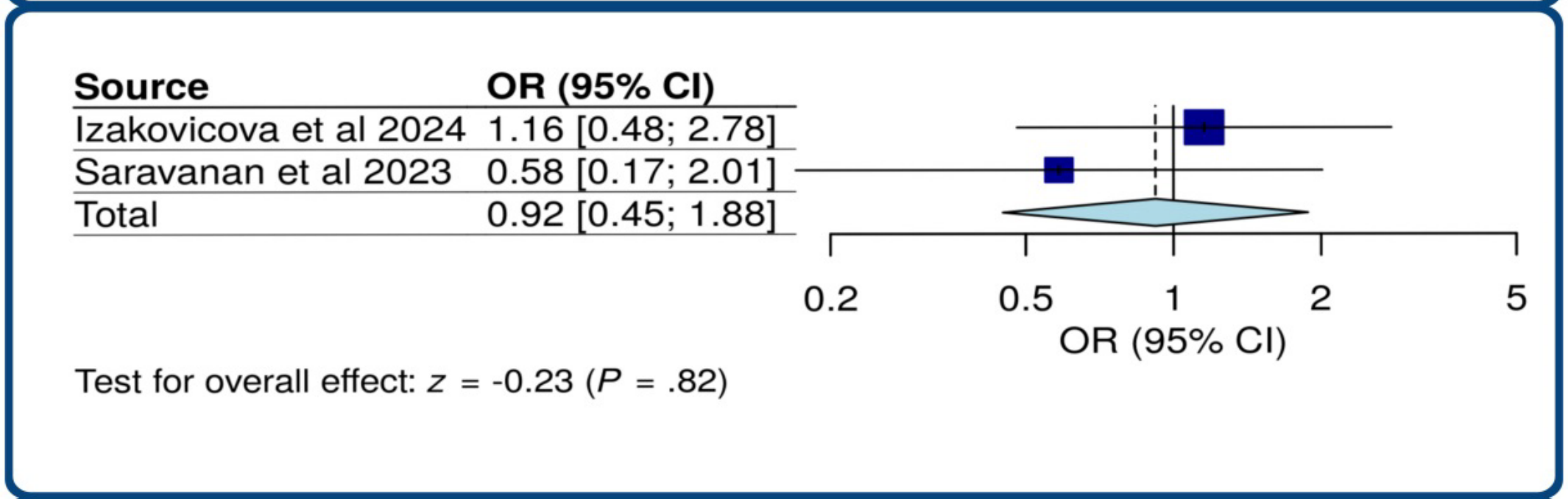

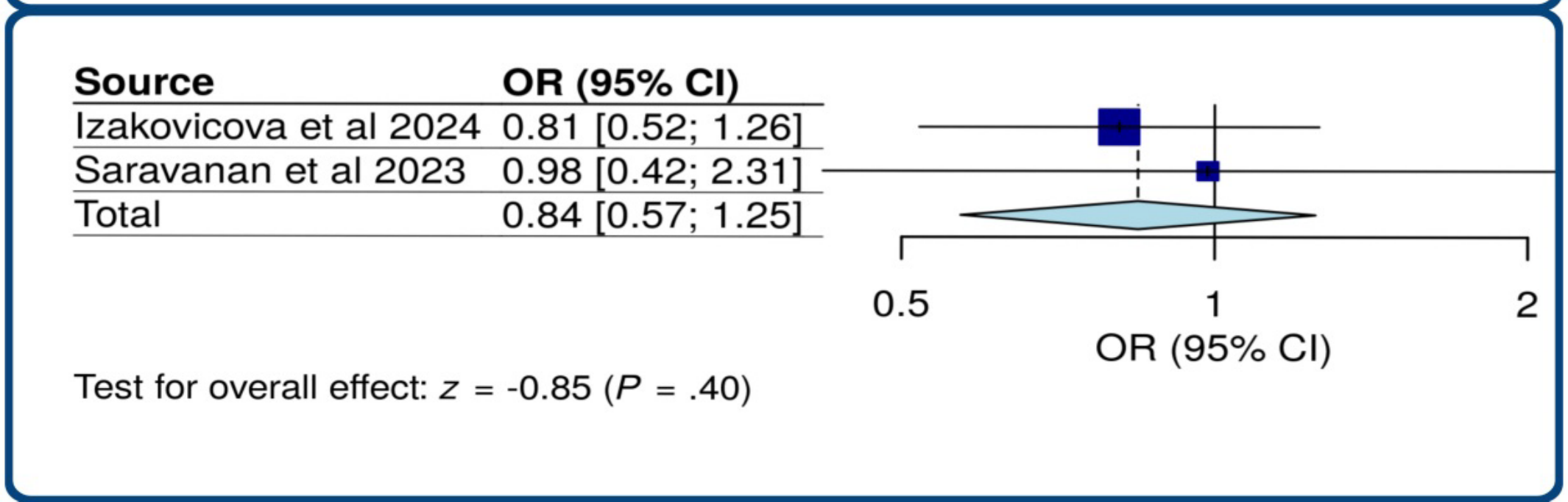

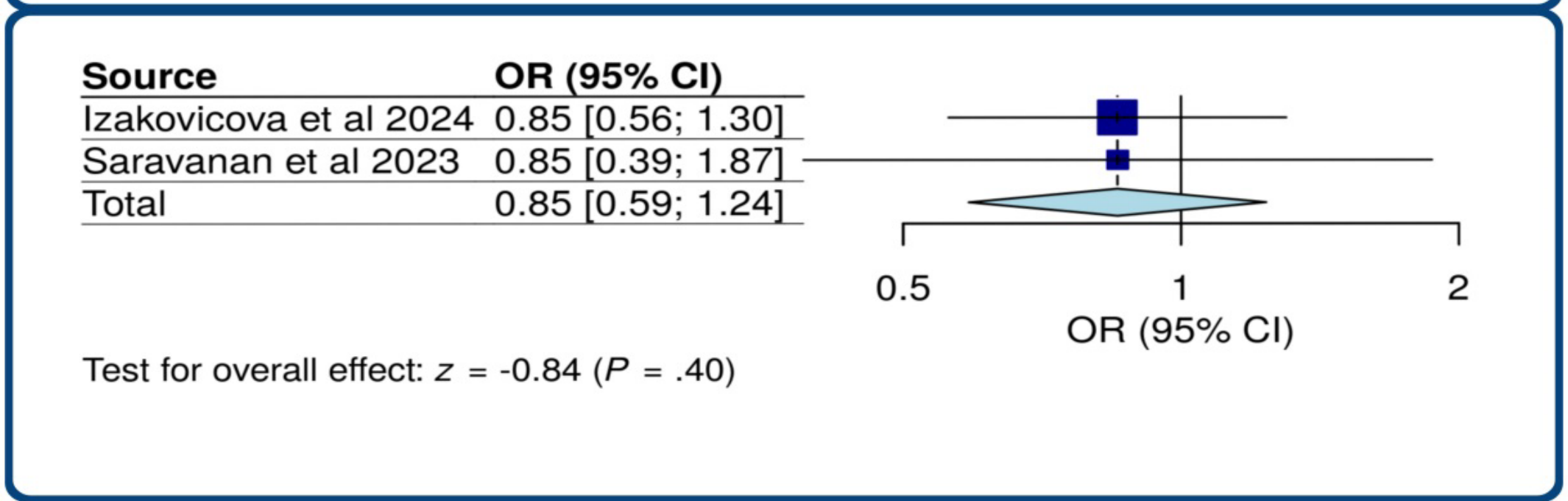

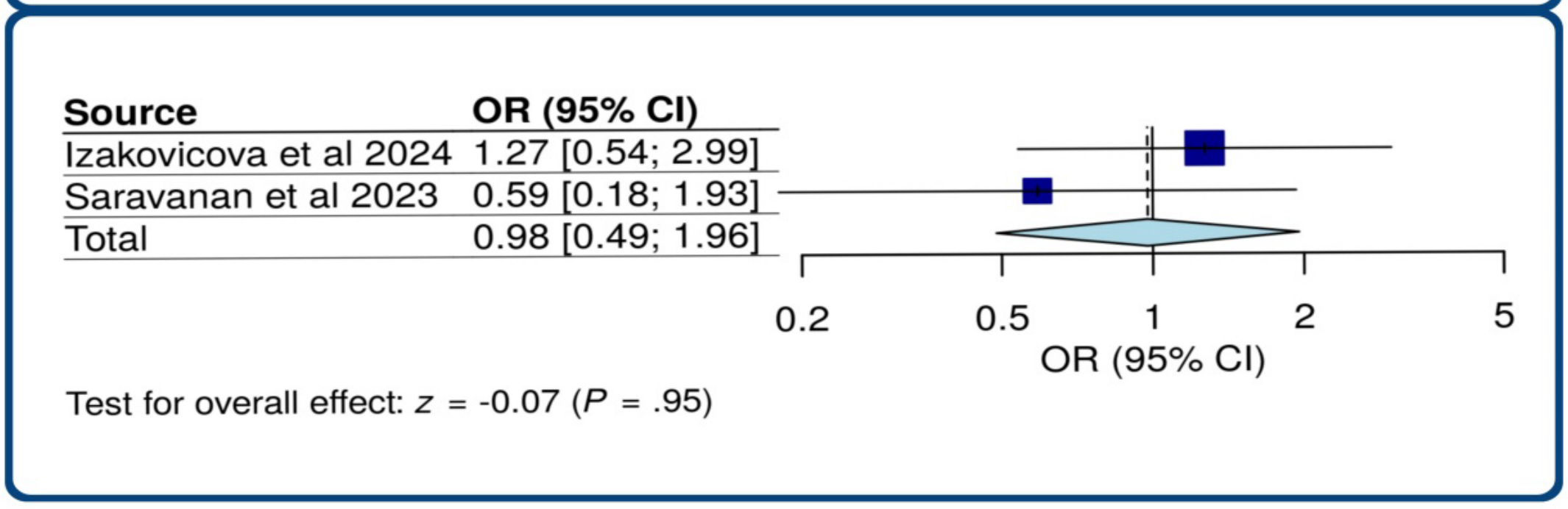

Supplement: Supplementary file 2 [file japid-18-153-s002.pdf]
