# Supplementary material for: Association of oxidative stress-related gst gene polymorphisms with periodontitis susceptibility: A systematic review and meta-analysis
Source: J Adv Periodontol Implant Dent. 2026 May 4;18(3):153–62. doi: 10.34172/japid.026.3866 (PMC13437218; doi:10.34172/japid.026.3866)
Supplement: Supplementary file 3 [file japid-18-153-s003.pdf]

Motahari and Neshanifard et al, J Adv Periodontol Implant Dent 2026;18(3):153-S3
doi:10.34172/japid.026.3866
https://japid.tbzmed.ac.ir

# Supplementary File 3

| Genetic polymorphism | No. of studies (participants) | Effect (OR, 95% CI) | Certainty (GRADE) | Reasons for downgrading |
|---|---|---|---|---|
| GSTM1-null | 6 studies (n ≈ 1,300) | OR = 2.94 (1.13–7.63) | Moderate | Risk of bias, some heterogeneity, imprecision |
| GSTT1-null | 5 studies (n ≈ 1,000) | OR = 0.64 (0.38–1.09) | Low | Small sample size, imprecision |
| GSTP1 (rs1695, all models) | 3 studies (n ≈ 500) | Initial ORs 1.5–2.0 across models; not robust after HWE-sensitive analysis | Low | Few studies, small samples, inconsistency |
